# A Brief Update on the Challenges and Prospects for Goat Production in Mexico

**DOI:** 10.3390/ani12070837

**Published:** 2022-03-25

**Authors:** Karen Tajonar, Carlos Antonio López Díaz, Luis Enrique Sánchez Ibarra, Alfonso Juventino Chay-Canul, Manuel Gonzalez-Ronquillo, Einar Vargas-Bello-Pérez

**Affiliations:** 1 Departamento de Medicina y Zootecnia de Rumiantes, Facultad de Medicina Veterinaria y Zootecnia, Universidad Nacional Autónoma de México, Av. Universidad 3000, Ciudad de Mexico 04510, Mexico; luis_enrique.si@comunidad.unam.mx; 2 Departamento de Economía Administración y Desarrollo Rural, Facultad de Medicina Veterinaria y Zootecnia, Universidad Nacional Autónoma de México, Av. Universidad 3000, Ciudad de Mexico 04510, Mexico; clopezd@unam.mx; 3 División Académica de Ciencias Agropecuarias, Universidad Juárez Autónoma de Tabasco, km 25. Carretera Villahermosa-Teapa, R/A La Huasteca, Villahermosa 86280, Mexico; alfonso.chay@ujat.mx; 4 Facultad de Medicina Veterinaria y Zootecnia, Universidad Autónoma del Estado de México, Av. Instituto Literario 100, Toluca 50000, Mexico; mrg@uaemex.mx; 5 Department of Veterinary and Animal Sciences, Faculty of Health and Medical Sciences, University of Copenhagen, Grønnegårdsvej 3, DK-1870 Frederiksberg C, Denmark

**Keywords:** goats, production, dairy, meat, Mexico, welfare, sustainability

## Abstract

**Simple Summary:**

Today, globally, there is a need for animal protein products. Goats are a viable option as they can transform feed to high-quality foods. In Mexico, information on goat production is scarce and documenting goat production challenges and future perspectives could be of great value, not only for Latin America, but also for international players—including those from farms, industry, and academia. The main challenges are correlated at different levels, where economic, social, and environmental issues are mixed and are closely associated with goat health and welfare. Newly available farming technologies could be an option that should be explored. Mexican goat farming systems will need to look at animal, social, and environmental factors to promote sustainable production systems.

**Abstract:**

In Mexico, information on goat production is scarce and documenting goat production challenges and future perspectives could be of great value, not only for Latin America, but also for international players—including those from farms, industry, and academia. Therefore, the objective of this review is to provide current knowledge on goat production systems in Mexico and discuss current challenges and future perspectives for this animal production sector. In Mexico, more than 70% of goats are produced under extensive production systems in arid and semi-arid areas and roughly 25% are produced in intensive or semi-intensive systems. Main breeds are French Alpine, Saanen, Toggenburg, LaMancha, Nubian, Boer, and their crosses. The main challenges are correlated at different levels, where economic, social, and environmental issues are mixed and are closely associated with goat health and welfare. Newly available farming technologies could be an option that should be explored. Mexican goat farming systems will need to look at the animal, social, and environmental factors to promote sustainable production systems.

## 1. Introduction

Globally, goat production has played an important role in rural areas as these animals can adapt easily to different environmental conditions and convert their feed into high-protein food sources such as meat and milk [1,2]. Goat whole-fresh milk is mainly produced in Asia (58.7%), followed by Europe (15.4%), Africa (21.9%), and the Americas (4%), with India as the main goat milk producer (5.4 million tons) [3]. Goat meat is mostly produced in Asia (72.4%), followed by Africa (23.5%), the Americas (2.2%), Europe (1.5%), and Oceania (0.4%), with China being the main goat meat producer at almost 1.6 million tons [3]. In addition, Asia also dominates production for goat skin, edible offal, and fat [3].

In Mexico, goats are mostly produced for meat and dairy production. The productive purpose (milk or meat) is found in different production systems and those vary between geographical areas. For example, meat production includes the traditional *cabrito* in Nuevo León, the *birria* in Jalisco and Zacatecas, the *mole de caderas* in Puebla, Guerrero, and Oaxaca, the *barbacoa* in the State of Mexico, Hidalgo, and Mexico City. Goat milk is used for manufacturing *cajeta* (sweet soft caramel), fresh and aged cheeses, and milk sweets, mainly in the center and northeast of Mexico [4].

Today, globally, there is a need for animal protein products. Goats are a viable option as they can transform feed to high-quality foods. In Mexico, information on goat production is scarce and documenting goat production challenges and future perspectives could be of great value, not only for Latin America, but also for international players—including those from farms, industry, and academia. Therefore, the objective of this review is to provide current knowledge on goat production systems in Mexico and provide detailed discussion on current challenges and future perspectives for this animal production sector.

The following sections are designed to provide a clear picture of current goat production in Mexico. In these sections, some historic facts will be discussed, along with a description of the main production systems and details on the main issues related to health, welfare, and environmental impacts. Lastly, current and future challenges will be discussed.

## 2. A Glance at the Origin of Goat Production in Mexico

The Spanish brought goats to Mexico more than 500 years ago. These animals were integrated into the Mexican livestock systems without major genetic improvements, which resulted in native or *criollo* animals [5]. Initially, the breeds brought from Spain included *Murciano-Granadina* (named Granadina in Mexico) and other breeds such as *Blanca Celtibérica* [6].

**Figure 1:**
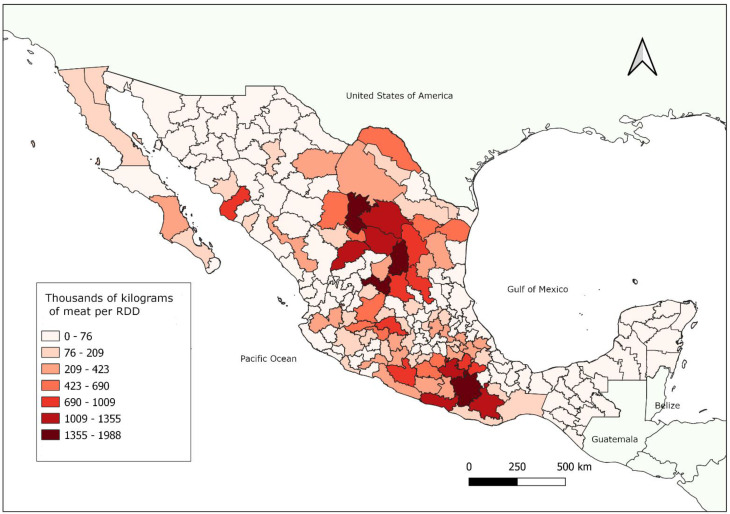
Distribution of goat meat production in Mexico by Rural Development Districts (RDD or DDR for its acronym in Spanish). There is an important concentration of systems (mostly extensive or semi-intensive) related to meat production in north and south Mexico. The main products are known as *cabrito* and *mole de caderas*. The Secretariat of Agriculture and Rural Development divides Mexico into 191 districts, according to geographic, social, and environmental factors that promote connection between the state, local farmers, and municipalities.

In the last 50 years, farmers started to import pure-breed goats (does and bucks) to improve their own flocks and offspring [7]. In this sense, breeds such as Alpine, Nubian, Saanen, and Toggenburg have a notable presence in the central and northern areas of Mexico. In 1980, goat breeding research officially started with comparison studies on pure breed and crossbreed animals, involving local goat genetics and goats from specialized breeds imported from the USA and using data from a large goat-breeding center in Tlahualilo, Durango, located in Northern Mexico [8]. Today, the goat breeds recognized by the National Association of Registered Goat Cattle Breeders (Asociación Nacional de Criadores de Ganado Caprino de Registro, ANCGCR) are French Alpine, Saanen, Toggenburg, LaMancha, Nubian and Boer [9]. For meat production, Nubian and, most recently, Boer goats have been used to improve growth traits, mainly under extensive management conditions distributed mostly in the northern and southern Mexico (Figure 1). Even though there are a few herds of purebred Nubian and Boer goats, bucks of both breeds have been used in crossbreeding programs with “*criollo*” and Nubian does for meat production improvement [8].

## 3. Goat Production Inputs on Mexican Economy

In Mexico, most goat farming is dual-purpose (meat and milk) and yearly, around 39,937 tons of meat and 161,901 tons of milk are produced [3]. In Mexico, more than 70% of goats are produced under extensive production systems in arid and semi-arid areas and roughly 25% are produced in intensive or semi-intensive systems [10]. Given the fact that most production is performed under extensive systems, it is difficult to obtain official records and therefore, the number of animals produced and consumed in each region of Mexico is still not available. It is estimated that the annual *per capita* consumption of goat meat is 0.4 kg, of which 2.1% is from imported meat. It is important to mention that a high percentage of goats are slaughtered and consumed by farmers and their families, and thus, the official data may not be so accurate [11].

## 4. Goat Production Systems

Goat production systems in Mexico can be divided into three major types: extensive, semi-extensive, and intensive [12]. However, some small variations within each system can be found depending on the country’s region.

In Mexico there are 494,000 goat farms and approximately 1.5 million Mexicans are dedicated to goat farming as a complementary productive activity. Most of these correspond to extensive systems [11], widely distributed in arid and semi-arid areas of Mexico [7]. This system is characterized by no or little use of technology and uses large land areas in which goats rotationally graze or are reared through transhumance. Under extensive conditions, animals are mostly fed on shrubs and any available forage [7].

**Figure 2:**
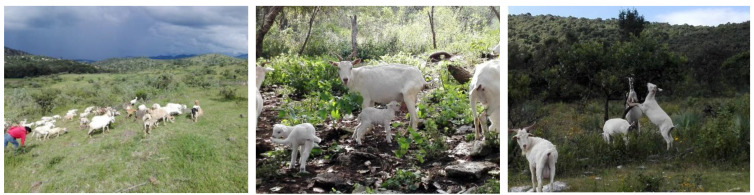
Transhumance systems in southern Mexico are distributed in dry, semi-arid and mountainous large areas (**left**). In this system, the shepherd herds the goats from coastal regions to mountainous regions such as Tehuacán, Puebla or to Huajuapan de León, Oaxaca. These animals are raised for the annual festival of *Mole de caderas* celebrated every autumn. One of the main breeds used in this system is the Mexican Pastoreña Goat (*Blanca Pastoreña*) (**middle**), which is well-adapted physiologically and anatomically to graze and browse in harsh environmental, orographical, and climate conditions (**right**).

There are three subsystems related to the extensive system that are based on the use of land for grazing and they are important for food security in rural areas. The first subsystem is free grazing, with greatest distribution in rural areas of Mexico, and in which animals graze crop residues. The herds are heterogeneous in terms of number of animals and mixtures of species, mainly with cattle and sheep. The second system is grazing with night confinement, in which the sale of dairy products such as cheese and animals are the main source of income for farmers. In this system, there is great consideration for the use of preventive medicine as well as some principles of food safety. The third subsystem is transhumance, which is characterized by goat migration between different regions of southern Mexico, from spring to mid-autumn. In this system, herds of hundreds to thousands of animals are reared mainly for meat, with no infrastructure and poor management practices [13]. In transhumance systems, the herds are composed of crossbreeds and a local goat breed known as Mexican Pastoreña. This local breed is known for its ability to travel long distances and its successful adaptation to the environmental conditions of that geographical area, which is composed of many mountains. The transhumance system has important biological value and constitutes an important economic resource for Mexico, as goats in this system are well-adapted to harsh conditions [2] (Figure 2).

**Figure 3:**
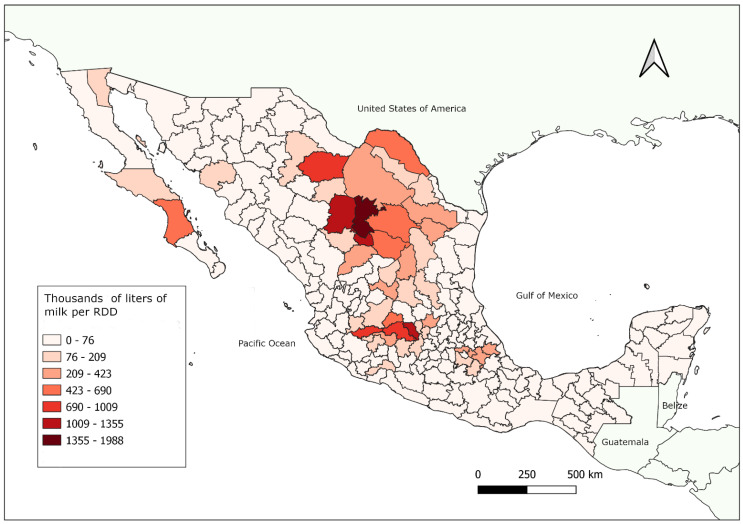
Distribution of goat milk production in Mexico by Rural Development Districts (RDD, or DDR for its acronym in Spanish). Notably, milk production under semi-intensive and intensive systems is mainly in central and north Mexico. These systems are near large cities, have easy access to technical support from veterinarians, and have close access to points of sale. The Secretariat of Agriculture and Rural Development divides Mexico into 191 districts, according to geographic, social, and environmental factors that promote connection between the state, local farmers, and municipalities.

**Figure 4:**
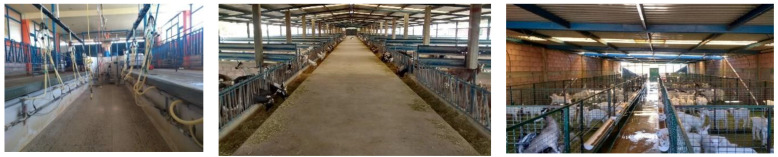
Intensive goat farming system from “*Granja Puente Colorado*”, located in Guanajuato of central Mexico and incorporated in the Guanajuato´s United Goat Farmers Association. This farm has been performing genetic improvements on different pure breeds. The main income comes from selling live animals, which is followed by fresh milk sales. As shown in the pictures (milking parlor (**left**), pens for adult goats (**middle**), and kid raising (**right**)), there has been great investment put into infrastructure and biosecurity to preserve animal welfare and health.

Intensive systems are mostly found in north and central Mexico (Figure 3), and goats are bred mainly for milk production and dairy product manufacturing (Figure 4). In this system, there is no grazing, animals receive total mixed diets (forage and concentrate), and compared to other systems, farmers have large investments in animal housing and farm infrastructure. In this system, farms use technologies for genetic improvement. In intensive systems—compared to other systems—regional farmers are well-organized and often receive technical support [14] from veterinary consultants, either through government programs or payment from the livestock associations.

The semi-intensive system represents a combination of the previous systems, where animals go out to controlled areas for grazing for some hours every day. In this system, there is a greater investment in housing compared to the extensive system, and goats receive high quality forage and/or concentrate as feed supplementation [11].

The farmer generally sells live animals to the local market for *barbacoa* or goat *birria* manufacturing, or as part of the breeding herd [15]. It is estimated that 58% are sold as weaned kids, followed by fattened animals at 20%, while 17% as used for self-consumption and 5% as breeding stock. Fattened or culled females are sold, but at lower prices. It is important to consider that extensive systems of goat production are based on traditional rearing, where goats are used for self-consumption.

### 4.1. Health

Today, the presence of goat dairies is increasing near urban centers. Health-related challenges include endemic goat diseases such as *Brucella mellitensis* and *Chlamydia spp*. [12]. Other challenges are the lack of veterinary and extension advice as well as diagnostic laboratory services (i.e., lentivirus detection) [16] 

In Mexico, consumer demand for goat milk products such as *cajeta*, cheeses, soaps, and cream has increased in recent years [17]. However, several production systems in Mexico are under extensive conditions and thus production remains low, but there are very few efforts for improving management and production outputs. Also, one of the challenges for Mexican dairy goat production is prevention of diseases such as brucellosis, mastitis [18], and paratuberculosis [19].

Brucellosis is characterized by the presence of abortions, which leads to reproductive problems and a considerable decrease in milk production (2 to 3 lactations), causing great economic losses in Mexican dairy goat production. Currently, the National Service for Agrifood Health, Safety and Quality (SENASICA) has a national campaign against brucellosis in animals, which aims to control and eradicate brucellosis in cattle, goats, and sheep. In areas with low prevalence, different strategic actions are carried out, such as the slaughter of positive animals, vaccination of infected herds, and diagnosis of disease-free herds. In medium- and high-prevalence areas, the strategy is mass vaccination [20].

In small ruminants, the prevalence of subclinical mastitis is 5% under a well-managed system [21], and the annual incidence of clinical mastitis is generally 5–10% [22,23]. Mastitis in small ruminants is generally a chronic and contagious infection and somatic cell count represents a valuable tool for prevalence assessment [21]. In Mexico, mastitis control in dairy systems is one of the major economic challenges, but economic losses can be reduced by adopting effective management and control programs. In western Mexico, bacteriological identification revealed the presence of *S. aureus*, *S. agalactiae*, *Corynebacterium* spp., and coliform bacteria [24]. In central Mexico, there have been efforts to characterize mastitis-related pathogens; the bacterial genera most frequently isolated in goat milk were coagulase-negative *Staphylococcus*, mainly *S. epidermidis* [25].

Paratuberculosis (PTB) or Johne’s disease is a chronic infectious disease caused by the *Mycobacterium avium* subspecies of paratuberculosis and is characterized by chronic and infectious granulomatous enteritis that produces progressive emaciation and ultimately the death of adult animals [26]. It can be transmitted through food, contaminated water, milk, or colostrum. In addition to this, in on-farm conditions, these bacteria survive at high temperatures, which is why it is mandatory under Mexican law (NOM-091-SSA1 1994) for all dairy products to be produced with pasteurized milk [27]. To establish the importance of the distribution of this disease in Mexico and its impact on production, a cross-sectional epidemiological study was performed in Puebla (central Mexico), comprising nine municipalities in *Libres* and ten in the *Mixteca Poblana*. A total of 58 males and 840 females were sampled, being 28.0% and 27.5% seropositive, respectively. From those animals, 252 were determined serologically positive for paratuberculosis. The apparent individual prevalence in the goat population was 28.06%, and in 100% of the municipalities studied, at least one positive herd was found [28]. Since this disease is a herd problem, biosecurity procedures and timely diagnoses should be considered for prevention. Measures such as detection and isolation of animals with clinical signs, such as low body condition and/or decreased milk yield, help in determining an accurate diagnosis [29]. Farmers and animal caretakers should be aware that the risk of higher prevalence of diseases increases with overcrowding, and therefore, general cleaning and disinfection of pens should be considered as a preventive measure for reducing risk of infections in intensive production systems [19]. Ideally, these measures should be adapted to other production systems.

### 4.2. Welfare

Sensitivity towards the use of farm animals for food production is increasing in Latin American countries such as Mexico, Chile, and Brazil [30,31]. In Europe, livestock welfare is among the top three issues that European consumers want to know more about, after safety and quality of foods, and the effect of agriculture on environmental and climate change [32,33,34]. There is evidence to believe that consumers in Mexico are moving in the same direction [35].

Regarding the increasing market for dairy goat production, which relies mostly on intensive systems, Lu and Miller [12] commented on the need to promote natural goat behavior and for this consideration to be shared among stakeholders. In this sense, there is a big challenge in intensive systems to provide environmental enrichment (i.e., housing improvement) that promotes natural behaviors. On the other hand, farmers in extensive conditions that have similar environmental conditions to that of wild goats face other issues; for instance, the difficulty in accessing technical and veterinary support and selling their products to urban markets.

In Mexico, goat-feeding strategies are not fully aligned with nutritional requirements. In this sense, feeding systems must ensure that nutrient requirements are met, but also allow natural ingestion behaviors, wherever and whenever possible. Recently, some research efforts have been made to improve dietary energy supply in dairy goat diets. For example, in central Mexico, whole seeds from different oilseeds, such as sunflower and linseed, have been included in grass silage-based diets [36], while calcium soaps from canola and safflower oils [37] have also been included in goat diets without negative effect on overall nutrient intake and digestibility.

Newer generations of consumers are increasingly sophisticated and willing to pay more for organic products to promote environmental sustainability and animal welfare [38,39]. In Mexico there are some research efforts trying to understand welfare issues at dairy goat farms. For example, in central Mexico, Salas Silva et al. [40] obtained comparative results between semi-intensive and intensive farms by using an integrated protocol of resource-based indicators and a set of animal-based indicators and concluded that there were better levels of welfare in semi-intensive farms where animals had access to grazing areas for most of the day. Although the authors mentioned that there are opportunities for improvement, nutritional issues were most preponderant.

Given that most goat populations in Mexico are in small-scale farms [41] under extensive and semi-intensive systems, feeding and parasitic diseases are common and should be closely monitored to improve the animals’ productive life [40]. On the other hand, in intensive systems, high animal density, no grazing, and lack of exercise leads to locomotion problems, and it has been recommended that preventive programs using hoof trimming and regular cleaning of bedding material be developed [40]. Regardless of the production system, Guatteo et al. [42] proposed refinement of some management practices in farm animals, such as suppressing specific invasive practices (e.g., avoiding dehorning and castration), substituting a management with a less painful procedure, or if those options cannot be performed (e.g., perform disbudding instead of dehorning), then soothing of pain should be considered (e.g., disbudding and castration using analgesics). Under the different types of production systems, goats can experience several stressing situations that could lead to decreased production. Those could range from aversive/harsh handling [43], high stocking densities [44,45], heat stress [46], constant changes in individuals between groups [47], and hoof lesions or overgrowth [48], to disbudding [49], dehorning, [50] and castration procedures [51]. In Mexico, the importance of these factors has not been reported and research studies exploring the aforementioned factors are needed.

In Europe, the Sm@ll Ruminant Technologies Platform [52] and the H2020 TechCare project [53] are research programs designed to share knowledge and improve production technologies for sheep and goats. Together, these grants account for almost 8 million Euros, reflecting the importance of these types of production systems in European public policies. Both projects aim at improving production efficiency, where all actors (researchers, farmers, and advisors) in the chain of production work together to improve awareness of available technologies (Precision Livestock Farming, PLF), showing their potential and economic benefits. These technologies are based on the use of PLF tools such as thermometers, weighting scales, video cameras, drones, and accelerometers, which help in obtaining data on productive traits, environmental conditions, and aid real-time goat/sheep behavior analysis for welfare and health monitoring. Mexico has the geographical conditions to lead this PLF field in Latin America, developing and using technologies that could help to make timely diagnoses and then preserve animal health.

### 4.3. Environmental Impact

Livestock has historically been subject to stigmatization, mainly due to its association with extensive systems linked to land dispossession and environmental degradation [54]. However, in general, it is recognized that grazing is one of the most sustainable production systems on the planet and plays a major role in safeguarding ecosystems and biodiversity in natural grasslands and rangelands [55]. Goats have great adaptability to extreme climatic conditions, regularly related to drought areas, as well as a greater capacity to convert feed into milk and meat with a value/quality superior to that of other domestic ruminants [56,57]. In Mexico, most of the goat production systems are located in these geographical regions. While goats have been linked to deforestation, the deterioration of the environment comes from a lack of or little sustainable management of natural resources [58]. A suggested strategy proposes reproductive management in order to optimize goat life cycle and also their emissions intensity of greenhouse gases [59], for example, via efficient timing of mating, births, and lactation in relation to the seasonal availability of fodder.

In an ideal sustainable farm, various factors must be considered to reach a point of equilibrium. Among these, Battaglini et al. [60] mentioned three axes of sustainability:

(1). Economic: degree of technification, administration/management of farms, the length of the production chain and quality of products, labeling (added value), direct sales and agro-ecotourism, use of endemic/local breeds, and food self-sufficiency.
(2). Social: intergenerational succession, professional training, recreational tourism, and animal welfare.
(3). Environmental: biodiversity, visual value and maintenance of the landscape, fire risk, soil erosion, greenhouse gas (GHG) emissions, and carbon sequestration.

The sustainability of any goat production system will depend on the 3 previous factors, while Peacock and Sherman [61] added the institutional factor, which involves skilled support services from the state and farm/governmental organizations.

Greenhouse gas emission and dairy waste disposal could be among the most important environmental concerns in dairy goat operations in the Americas [62]. Intensive and extensive dairy goat production systems have different GHG profiles. Methane emission is generally lower in dairy goats raised in a confinement system, where they are fed with more digestible diets high in concentrate [63]. Dairy goat grazing pastures can produce more methane, because the animals are consuming more fiber and less digestible diets. However, when their manure is used for plant growth in pastures, more carbon is removed from the atmosphere and is sequestered [64]. Experience with dairy cow waste treatment provides important lessons for dairy goat production. Although goat manure is easier to handle than cow manure, as farms get larger, it must be managed properly to minimize methane production, reduce odors, and to avoid contamination of water resources [62]. In Mexico, these lessons could be taken into consideration in dairy goat systems. In Mexico, some research has been done in dairy cattle [64,65], but this remains an unexplored field for goat production.

Interestingly, Gómez-Ruiz et al. [58] made a retrospective analysis on goat cheese production in a rangeland from arid areas, and recognized that under these conditions, organized farms by cooperatives promoted milk fat yields of around 6.8%. This is an example of how successful it is to organize farmers towards one single objective. In Mexico, the lack of farmer organization is a factor that needs to be improved.

**Figure 5:**
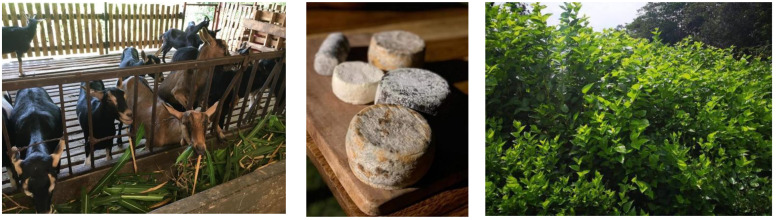
Sustainable farm “Don Nelo” located in eastern Mexico in Veracruz (**left**). This region is characterized by mountains and subtropical climate. A combination of elements in the system, from diversified production (spreadable and aged cheeses (**middle**), figs stuffed with cheese, coffee, live animal sales, manure, farm training and gastronomic workshops), agroforestry technologies and agro-ecological practices, to low-cost feed such as *Morera (Morus alba*) (**right**). This system results in higher productivity and profitability for the producer and promotes circular economy at a local level.

Torres Rivera and Palma García [66] made a review of the multifunctional farm “Don Nelo” (Figure 5). This type of system is focused on protection and enhancement of natural resources used for environmental services, product diversification with regional and extra-regional sales, local job creation and agrotourism, self-production of forage sources, and the use of local agro-industrial by-products.

## 5. Challenges and Opportunities

**Figure 6:**
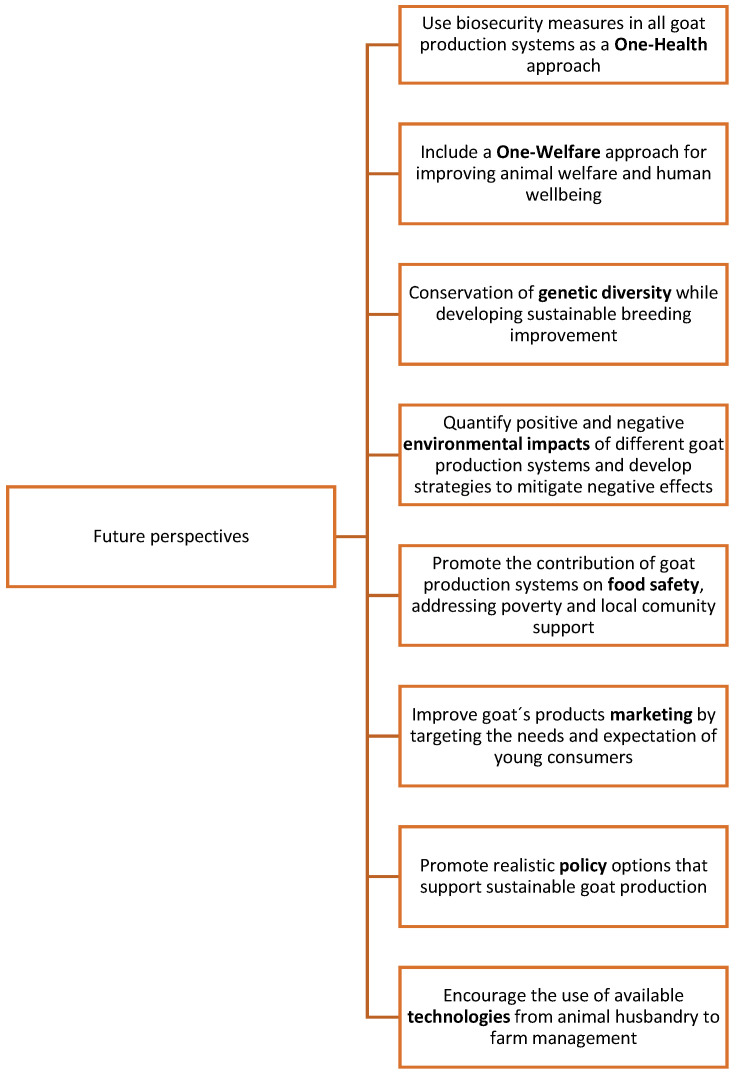
Future perspectives for Mexican goat production development.

The following section will describe some of the challenges and opportunities that the authors consider to be pivotal for the development of Mexican goat production. These consider different angles (health, welfare, genetic diversity, and environmental impact), and dimensions (goats and humans) that should be studied to shape more efficient goat production systems in Mexico; summarized in Figure 6.

### 5.1. Health, Welfare and Genetics

Technical training for staff on different husbandry practices is needed to prevent health problems and preserve animal welfare [67]. In this sense, there is a need to develop an integral welfare-monitoring program, considering animal welfare as the transversal axis in every goat husbandry practice. On the other hand, developing genetic selection of dairy goats to match productive traits as well as their ability to cope with the specific environmental conditions of Mexico is vital to ensure a sustainable development of this production system. For example, the high fat content is a productive trait that deserves more attention [58].

### 5.2. Environmental Impacts

There is a need for management of communal lands that addresses the technical dimensions and governance of territories, aimed at avoiding environmental deterioration, promoting the rational use of natural resources, and increasing carbon fixation capacity of rangelands in compliance with national regulations focused on sustainable production systems [61]. In addition, Mexican goat production should consider reducing the carbon cost and footprint of goat products through efficient reproductive programs, coordinated with optimized nutritional management and maximized neonatal survival, thereby increasing productivity [59].

### 5.3. Food Safety and Marketing

It is important to encourage regional sales and consumption to improve nourishment for rural families and build circular economy [7], as well as promote the added value of existing goat products due to their nutritional benefits for humans [68] and their potential for sustainable production [61]. It is relevant to consider that the transformation and marketing of products (i.e., milk to cheese) within farms would be more lucrative than selling to collectors or middlemen [58]. One approach could be to improve farmer organization, production, and commercialization to improve economic revenues from goat products such as fluid milk [58].

### 5.4. Policy, Territory and Gender Equalty

The United States–Mexico–Canada Agreement that entered into force on 1 July 2020 [68] offers a great opportunity to develop Mexican goat production, by reaching different market niches. Also, at the national level, collaboration between regional producers and/or organizations is necessary to agree on regulations and prices [12]. One challenge for goat production is the existing fragmentation of Mexican territory due to organized crime [69,70]. The largest numbers of goats are found in areas that are under organized crime control, and therefore, governmental aid is scarce or not available. Another challenge is to recognize the enormous role of women in rural families. The need for some family members to migrate, either temporarily or permanently, results in many women taking care of the small-scale herds [71].

### 5.5. Husbandry Technologies and Farm Management

It is pivotal to promote the research, development, and transfer of national PLF technologies for goat production. Promoting academic and governmental mechanisms will develop multidisciplinary research that can identify the opportunities and limitations of different goat production systems. In terms of management practices, milk can be sold after kid weaning in systems focused on meat production for extra income or self-consumption.

## 6. Final Remarks and Reflections

Goat production systems have a socio-economic impact in Mexico. Two major strengths of Mexican goat production are its diversity of production systems and its variety of goat products. Consequently, goat farming research should consider productive aspects as well as social implications. In Mexico, it is estimated that more than half of the production units are managed by farmers around 50 years old, with inherited production systems [15]. Therefore, an important approach to be considered is a focus on young people that provides them with knowledge that will prepare them for the technical challenges of the goat sector.

Because extensive systems are distributed throughout Mexico, organized groups of farmers working together are needed to increase marketing and commercialization of goat products, with clear missions and visions. Collaborative work should be promoted between farmers, veterinary assistance, and technical professionals. This will improve farmer welfare and ensure continuity of farmer family businesses. In addition, promotion of local consumption of goat products is important as it has great potential on preventing human malnutrition.

Goats play an important role as a provider of eco-services by way of conservation of natural resources. Normally, in extensive systems, goats are fed on plants and/or forages with high contents of secondary metabolites such as tannins and saponins that could promote the formation of unique compounds in goat products. This represents an opportunity to obtain goat products that could be commercialized in niche markets where consumers are looking for animal products with some functionality.

Lastly, geopolitically, Mexico is considered in North America and has trade agreements with Canada and the United States of America; therefore, these agreements should be used in favor of better commercialization of goat products. Perhaps one approach lies in promoting the market for niche products (such as those produced via transhumance), as happens in Europe with dairy products produced in high mountains in the Alps.

## Data Availability

The data presented in this study are available on request from the corresponding author.

## References

[1] Castel J., Ruiz F., Mena Y., Sánchez-Rodríguez M. (2010). Present situation and future perspectives for goat production systems in Spain. Small Rumin. Res..

[2] Domínguez M., De La Rosa J.D.P., Landi V., De La Rosa J.P., Vazquez N., Martinez A., Fuentes-Mascorro G. (2018). Genetic diversity and population structure analysis of the Mexican Pastoreña Goat. Small Rumin. Res..

[3] FAO FAOSTAT. CC BY-NC-SA 3.0 IGO. https://www.fao.org/faostat/es/#data/QCLF.

[4] Anzaldo-Montoya M. (2019). Entre la vulnerabilidad y la invisibilidad científica. Estudio sobre los aportes de las ciencias sociales a la investigación sobre ganadería caprina en México. Rev. Aliment. Contemp. Desarro. Reg..

[5] Tovar-Luna I. (2009). Goat Production in Mexico. Overview of the Industry and Its Production Practices. Proceedings of the 24th Annual Goat Field Day.

[6] Martínez Rojero R.D., Torres Hernández G., Martínez Hernández S. (2014). Caracterización Fenotípica, Productiva y Reproductiva de La Cabra Blanca Criolla Del “Filo Mayor” de La Sierra Madre Del Sur En El Estado de Guerrero / Phenotypic, Productive and Reproductive Characterization of the White Creole Goat of the “Filo Mayor” f. Nova Sci..

[7] Escareño L., Salinas-Gonzalez H., Wurzinger M., Iñiguez L., Sölkner J., Meza-Herrera C. (2012). Dairy Goat Production Systems: Status Quo, Perspectives and Challenges. Trop. Anim. Health Prod..

[8] Montaldo H., Torres-Hernández G., Valencia-Posadas M. (2010). Goat breeding research in Mexico. Small Rumin. Res..

[9] Asociación Nacional de Criadores de Ganando Caprino de Registro AC Estándares—ANCAPRINA. https://ancaprina.org.mx/estandares/.

[10] Mellado M. (1997). La Cabra Criolla En América Latina. Vet. Méx..

[11] Aréchiga C.F., Aguilera J.I., Rincón R.M., Méndez de Lara S., Bañuelos V.R., Meza-Herrera C.A. (2008). Role and Perspectives of Goat Production in a Global World. Trop. Subtrop. Agroecosystems.

[12] Lu C.D., Miller B.A. (2019). Current status, challenges and prospects for dairy goat production in the Americas. Asian-Australasian J. Anim. Sci..

[13] Ramírez J.M.P., Sánchez O.M., Ortiz B.R., Zaragoza R.J.L., Ricardi D.L.C.L.C., Fuentes-Mascorro G. (2014). Creole Goat “Pastoreña” Production System and Zoometric Measurement in Mixteca Oaxaca. Actas Iberoam. Conserv. Anim..

[14] Oliveros-Oliveros J., Morales-Arzate J.J., Andrade-Montemayor H. (2009). Productive Progress in a Goat Producers Association, “Caprinocultores Unidos De Guanajuato AC”, through a Technology Transfer System GGAVATT (Livestock Validation and Technology Transfer Group) (2001–2007). Trop. Subtrop. Agroecosystems..

[15] García-Bonilla D.V., Vargas-López S., Bustamante-González A., Torres-Hernández G., Calderón-Sánchez F., Olvera-Hernández J.I. (2018). Goat Production for Meat in the Mountain of Guerrero, Mexico. Agric. Soc. Desarro..

[16] De la Luz-Armendáriz J., Ducoing-Watty A.E., Ramírez-Mendoza H., Gómez-Núñez L., Tufiño-Loza C., Cabrera-Domínguez E.M., Díaz-Aparicio E., Rivera-Benítez J.F. (2021). Prevalence, Molecular Detection, and Pathological Characterization of Small Ruminant Lentiviruses in Goats from Mexico. Small Rumin. Res..

[17] Santos-Lavalle R., Flores-Verduzco J.J., Cervantes-Escoto F., Salas-González J.M., Sagarnaga-Villegas L.M. (2018). Opportunities for Goat Farmers in Guanajuato, Mexico, in the Marketing of Fine Cheese|Oportunidades Para Caprinocultores de Guanajuato, México, En La Comercialización de Queso Fino. Rev. Mex. Cienc. Pecu..

[18] Hernández Hernández B. (2006). Producción de Leche de Cabra y Su Industrialización, Una Opción Para El Estado de Hidalgo. Bachelor’s Thesis.

[19] Guzmán C.C., Santillán M.A., Córdova D. (2016). Prevalence and possible risk factors for caprine paratuberculosis in intensive dairy production units in Guanajuato, Mexico. J. Vet. Med. Anim. Health..

[20] Servicio Nacional de Sanidad, I. y C.A. Brucelosis En Animales|Servicio Nacional de Sanidad, Inocuidad y Calidad Agroalimentaria|Gobierno|Gob.Mx. https://www.gob.mx/senasica/acciones-y-programas/campana-nacional-contra-la-brucelosis.

[21] Bergonier D., de Crémoux R., Rupp R., Lagriffoul G., Berthelot X. (2003). Mastitis of dairy small ruminants. Vet. Res..

[22] Contreras A., Paape M., Miller R. (1999). Prevalence of subclinical intramammary infection caused by *Staphylococcus epidermidis* in a commercial dairy goat herd. Small Rumin. Res..

[23] Silanikove N., Leitner G., Merin U., Prosser C. (2010). Recent advances in exploiting goat’s milk: Quality, safety and production aspects. Small Rumin. Res..

[24] Vázquez H.C., Jäger S., Wolter W., Zschöck M., El-Sayed A. (2013). Isolation and identification of main mastitis pathogens in Mexico. Vet. Med..

[25] Romero R.A.R., Martínez S.P.M., Olivares R.A.C., Ortíz V.E.E., Watty A.E.D. (2018). Identification of and antimicrobial resistance in bacteria causing caprine mastitis in three states and a city in Central Mexico under manual and mechanical milking conditions. J. Dairy, Vet.-Anim. Res..

[26] Estévez-Denaives I., Hernández-Castro R., Trujillo-García A., Chávez-Gris G. (2007). Detection of Mycobacterium avium subsp. paratuberculosis in goat and sheep flocks in Mexico. Small Rumin. Res..

[27] Hilario M.C., Puga C.D., Wrage N., Pérez-Gil R. (2010). Feeding goats on scrubby Mexican rangeland and pasteurization: Influences on milk and artisan cheese quality. Trop. Anim. Health Prod..

[28] Maldonado É.P.G., Reynoso B.A., Flores M.A.S., Humara L.D.C.F., López D.C., Morales R.J., Aparicio E.D. (2017). Epidemiological situation of paratuberculosis in mainly goat regions of Puebla, Mexico. Quehacer Científico Chiapas.

[29] Resendiz G.P., Romero F.A., Pérez C.F., Núñez L.G., Hernández J.G., López E.H., González M.L., Álvarez F.M., López F.P., Aparicio E.D. (2021). Enfermedades infecciosas de relevancia en la producción caprina, historia, retos y perspectivas. Rev. Mex. Cienc. Pecu..

[30] Vargas-Bello-Pérez E., Riveros J.L., Köbrich C., Álvarez-Melo P.A., Lensink J. (2017). Chilean consumers’ perception about animal welfare in dairy production systems: Short communication. Anim. Prod. Sci..

[31] Vargas-Bello-Pérez E., Miranda-de la Lama G.C., Teixeira D.L., Enriquez-Hidalgo D., Tadich T., Lensink J. (2017). Farm Animal Welfare Influences on Markets and Consumer Attitudes in Latin America: The Cases of Mexico, Chile and Brazil. J. Agric. Environ. Ethic..

[32] Evans A., Miele M. (2008). Consumers’ Views about Farm Animal Welfare, Part II: European Comparative Report Based on Focus Group Research.

[33] (2016). European Commission, Directorate-General for Health and Food Safety, Attitudes of Europeans towards animal welfare: Report, European Commission. J. Eur. Union.

[34] (2021). European Parliament, Directorate-General for Parliamentary Research Services, Karamfilova, E., Animal welfare on the farm, ex-post evaluation of the EU legislation: Prospects for animal welfare labelling at EU level: European implementation assessment, European Parliament. J. Eur. Union.

[35] Miranda-de la Lama G.C., Estévez-Moreno L.X., Sepúlveda W.S., Estrada-Chavero M.C., Rayas-Amor A.A., Villarroel M., María G.A. (2017). Mexican consumers’ perceptions and attitudes towards farm animal welfare and willingness to pay for welfare friendly meat products. Meat Sci..

[36] Vargas-Bello-Pérez E., De Oca C.A.G.M., Salas N.P., Flores J.G.E., Bernal J.R., Robles-Jimenez L.E., Gonzalez-Ronquillo M. (2020). Productive Performance, Milk Composition and Milk Fatty Acids of Goats Supplemented with Sunflower and Linseed Whole Seeds in Grass Silage-Based Diets. Animals.

[37] Vargas-Bello-Pérez E., Robles-Jimenez L.E., Ayala-Hernández R., Romero-Bernal J., Pescador-Salas N., Castelán-Ortega O.A., González-Ronquillo M. (2020). Effects of Calcium Soaps from Palm, Canola and Safflower Oils on Dry Matter Intake, Nutrient Digestibility, Milk Production, and Milk Composition in Dairy Goats. Animals.

[38] Lu C.D. (2011). Nutritionally related strategies for organic goat production. Small Rumin. Res..

[39] Lu C., Gangyi X., Kawas J. (2010). Organic goat production, processing and marketing: Opportunities, challenges and outlook. Small Rumin. Res..

[40] Salas M.Á.S., Mondragón-Ancelmo J., Badillo M.D.R.J., Licea G.R., Napolitano F. (2021). Assessing dairy goat welfare in intensive or semi-intensive farming conditions in Mexico. J. Dairy Sci..

[41] Escareño-Sánchez L.M., Wurzinger M., Pastor-López F.J., Salinas H., Sölkner J., Iñiguez L. (2011). La cabra y los sistemas de producción caprina de los pequeños productores de la comarca lagunera, en el norte de méxico. Rev. Chapingo Ser. Cienc. For. Ambient..

[42] Guatteo R., Levionnois O., Fournier D., Guémené D., Latouche K., Leterrier C., Mormede P., Prunier A., Servière J., Terlouw E.M.C. (2012). Minimising pain in farm animals: The 3S approach—‘Suppress, Substitute, Soothe’. Animal.

[43] Baxter E.M., Mulligan J., Hall S.A., Donbavand J.E., Palme R., Aldujaili E., Zanella A.J., Dwyer C.M. (2016). Positive and negative gestational handling influences placental traits and mother-offspring behavior in dairy goats. Physiol. Behav..

[44] Chojnacki R.M., Vas J., Andersen I.L. (2014). The Effects of Prenatal Stocking Densities on the Fear Responses and Sociality of Goat (*Capra hircus*) Kids. PLoS ONE.

[45] Vas J., Chojnacki R., Kjøren M.F., Lyngwa C., Andersen I.L. (2013). Social interactions, cortisol and reproductive success of domestic goats (*Capra hircus*) subjected to different animal densities during pregnancy. Appl. Anim. Behav. Sci..

[46] Salama A., Caja G., Hamzaoui S., Badaoui B., Castro-Costa A., Façanha D., Guilhermino M., Bozzi R. (2014). Different levels of response to heat stress in dairy goats. Small Rumin. Res..

[47] Patt A., Gygax L., Wechsler B., Hillmann E., Palme R., Keil N.M. (2012). The introduction of individual goats into small established groups has serious negative effects on the introduced goat but not on resident goats. Appl. Anim. Behav. Sci..

[48] Christodoulopoulos G. (2009). Foot lameness in dairy goats. Res. Vet.-Sci..

[49] Thompson K.G., Bateman R.S., Morris P.J. (2004). Cerebral Infarction and Meningoencephalitis Following Hot-Iron Disbudding of Goat Kids. New Zealand Vet. J..

[50] Acharya M. (2018). Pain minimization during dehorning and disbudding in goats and goat kids. Bangladesh J. Vet.-Med..

[51] Ahmed S.A., Ahmed E.A. (2011). Behavioral Responses of Castrated Buck Kids at Different Ages By Using Different Methods of Castration. J. Anim. Sci..

[52] Small Ruminant Technology—Precision Livestock Farming and Digital Technology for Small Ruminants|SmaRT Project|Fact Sheet|H2020|CORDIS|European Commission. https://cordis.europa.eu/project/id/101000471/es.

[53] Integrating Innovative TECHnologies along the Value Chain to Improve Small Ruminant WelfARE Management|TechCare Project|Fact Sheet|H2020|CORDIS|European Commission. https://cordis.europa.eu/project/id/862050.

[54] Cavallotti Vázquez B.A., Ramírez Valverde B., Cesín Vargas A., Ramírez Juárez J., Pablos J. (2017). Globalización, Seguridad Alimentaria y Ganadería Familiar.

[55] Johnsen K.I., Niamir-Fuller M., Bensada A., Waters-Bayer A. (2019). A Case of Benign Neglect: Knowledge Gaps about Sustainability in Pastoralism and Rangelands.

[56] Navarrete-Molina C. (2020). The Ruminant Production Systems in the Comarca Lagunera, Mexico: Environmental Impact, Productive Trends, and Mitigation Strategies. Ph.D. Thesis.

[57] Sintori A., Tzouramani I., Liontakis A. (2019). Greenhouse Gas Emissions in Dairy Goat Farming Systems: Abatement Potential and Cost. Animals.

[58] Gómez-Ruiz W.J., Pinos-Rodríguez J.M., Aguirre-Rivera J.R., García-López J.C. (2012). Analysis of a goat milk cheese industry in a desert rangeland of Mexico. Pastor. Res. Policy Pract..

[59] Delgadillo J.A., Martin G.B. (2015). Alternative methods for control of reproduction in small ruminants: A focus on the needs of grazing industries. Anim. Front..

[60] Battaglini L., Bovolenta S., Gusmeroli F., Salvador S., Sturaro E. (2014). Environmental Sustainability of Alpine Livestock Farms. Ital. J. Anim. Sci..

[61] Peacock C., Sherman D. (2010). Sustainable goat production—Some global perspectives. Small Rumin. Res..

[62] Miller B.A., Lu C.D. (2019). Current status of global dairy goat production: An overview. Asian-Australasian J. Anim. Sci..

[63] Pragna P., Chauhan S.S., Sejian V., Leury B.J., Dunshea F.R. (2018). Climate Change and Goat Production: Enteric Methane Emission and Its Mitigation. Animals.

[64] Pineda G.S.H., Beltrán P.E.P., Benaouda M., García J.M.P., Nova F.A., Molina L., Ortega O.A.C. (2018). Pithecellobium dulce, Tagetes erecta and Cosmos bipinnatus on reducing enteric methane emission by dairy cows. Ciência Rural.

[65] Rendón-Huerta J., Pinos-Rodríguez J., Kebreab E., García-López J., Vicente J. (2018). Comparison of greenhouse gas emissions from Mexican intensive dairy farms. South Afr. J. Anim. Sci..

[66] Torres Rivera J.A., Palma García J.M., Palma García J.M., Cruz Uribe J.F. (2021). De La Agricultura Convencional a La de Tipo Multifuncional. Caso Granja “Don Nelo”. Tecnologías Sociales en la Producción Pecuaria de América Latina y el Caribe.

[67] Hempstead M., Lindquist T., Shearer J., Shearer L., Plummer P. (2021). Health and Welfare Survey of 30 Dairy Goat Farms in the Midwestern United States. Animals.

[68] United States-Mexico-Canada Agreement|United States Trade Representative USMCA|United States Trade Representative. https://ustr.gov/usmca.

[69] Gutiérrez-Romero R. (2015). Estimating the impact of Mexican drug cartels and drug-related homicides on crime and perceptions of safety. J. Econ. Geogr..

[70] Atuesta L.H., Pérez-Dávila Y.S. (2017). Fragmentation and cooperation: The evolution of organized crime in Mexico. Trends Organ. Crime.

[71] Sinn R., Ketzis J., Chen T. (1999). The Role of Woman in the Sheep and Goat Sector. Small Rum. Res..

